# Early-life pain alters excitability of corticotropin-releasing factor-expressing neurons in the central amygdala and stress-induced hypersensitivity during adolescence

**DOI:** 10.3389/fnbeh.2025.1653346

**Published:** 2025-11-26

**Authors:** Megan Tomasch, Emma Naess, Skyler McComas, Michael A. Burman

**Affiliations:** 1 Graduate School of Biomedical Science and Engineering, University of Maine, Orono, ME, United States; 2 Center for Excellence in the Neurosciences, University of New England, Biddeford, ME, United States; 3 School of Social and Behavioral Sciences University of New England, Biddeford, ME, United States

**Keywords:** neonatal pain, CRF neurons, central amygdala, chemogenetics, patch-clamp, electrophysiology

## Abstract

**Introduction:**

Neonatal intensive care units (NICUs) provide life-saving care for preterm and sick neonates, but many medical procedures are painful and stress-inducing. Even a routine NICU procedure, such as the “heel lancing” blood-draw procedure, is an acutely painful, repetitive manipulation that has lasting negative impacts on pain perception and anxiety responses. The intersection of nociception and negative affect occurs in a brain region called the central nucleus of the amygdala (CeA), and neurons expressing corticotropin-releasing factor (CRF) have been implicated in studies of both anxiety and pain.

**Methods:**

Using a two-hit model of trauma-induced pain vulnerability—where repetitive needle prickings occur during the first week of life (“our NICU model”), followed by a second stressor (e.g., fear conditioning) during adolescence—our lab has observed a mechanical hypersensitivity in rats that endured our NICU model that manifests only after fear conditioning. We have also observed changes to expression and activation of CeA-CRF neurons after the NICU-like experience with an acute increase followed by a lasting reduction in the number of CRF cells in the right CeA of adolescent male rats. However, the relationship between these changes and the observed behavioral outcomes remains unclear, as does the function of the remaining CRF cell population. We hypothesize that the remaining population of CRF-expressing CeA neurons are functionally altered by early life pain and stress and primed to respond more readily, such that vulnerability to stress-induced hypersensitivity is increased.

**Results:**

Through chemogenetic inhibition of the amygdala, or specifically CeA-CRF neurons, we demonstrate that development of stress-induced mechanical hypersensitivity after our NICU model is completely reversed through silencing the amygdala. Inhibiting only CeA-CRF neurons during fear conditioning led to a partial reversal of the hypersensitivity, suggesting that other populations of cells also play critical roles. Nevertheless, we demonstrate that the NICU-like experience results in a lasting hyperexcitability of CeA-CRF neurons during adolescence, confirming that this population is affected by the early life manipulations.

**Discussion:**

In all, this study suggests that CeA-CRF neurons may have pro-nociceptive properties that are exacerbated by early life pain and result in maladaptive responding to subsequent traumatic events.

## 1 Introduction

Early-life pain, such as that experienced through medical interventions in the neonatal intensive care unit (NICU), has been shown to alter developmental trajectories and have long-lasting repercussions. The plasticity of the brain in early (peri- and postnatal) development means it is particularly vulnerable (Fitzgerald, 2005). Experiencing pain during early development has been shown to have a negative impact on brain development, resulting in neurosensory impairments and poorer cognitive outcomes (i.e., lower IQ scores, language and attention deficits, behavioral problems, etc.) that impact individuals into adulthood (Williams and Lascelles, 2020). Furthermore, early-life pain has been shown to alter subsequent pain responses as early as term -age in infants born preterm, through adolescence, and into adulthood, with individuals often displaying enhanced pain sensitivity to additional insults (Schwaller and Fitzgerald, 2014; Lidow, 2002). Over the last 5 years, the US has seen a 13% increase from 2016 (8.7%) to 2023 (9.8%) in the percentage of newborn infants admitted to the NICU (Martin and Osterman, 2025), making the need to understand the long-term impacts of early-life pain increasingly important.

The neurobiological mechanisms through which early-life pain experienced in the NICU continues to impact individuals long term are difficult to study due to the invasive nature of the investigations necessary. Moreover, there is much variability among human patients [i.e., different gestational ages or other medical conditions/complications, as well as different racial and socioeconomic factors (Ravi et al., 2025)], making it difficult to determine causal factors if relying solely upon clinical data. As such, preclinical animal models of early-life pain and stress have become a key tool for understanding the long-term impacts of early-life adversity (Williams and Lascelles, 2020; Anand et al., 1999; Plotsky et al., 2005; Victoria and Murphy, 2016b). Animal models employing techniques intended to create early-life adversity and replicate trends observed clinically have successfully done so, but the wide range of outcomes or specific stipulations observed across studies means more investigation is still necessary. One model of early-life pain, “repetitive needle pricking,” replicates a routine blood draw procedure that would be experienced in the NICU by both preterm and full-term neonates, called “heel lancing,” through a series of small needle-pricks over the course of the first few days or weeks of development (Anand et al., 1999; Beggs et al., 2012; Davis et al., 2021, 2018, 2020; Davis and Burman, 2021; Zuke et al., 2019; Mooney-Leber et al., 2018; Moriarty et al., 2019). In studies replicating this experience, researchers typically observe pain hypersensitivity in later life (Anand et al., 1999; Beggs et al., 2012; Knaepen et al., 2013). Researchers interested in the impacts of neonatal surgery typically replicate the experience with a neonatal hind paw incision, typically followed by a subsequent incision in later life, as many children will also need additional surgeries as they develop (Beggs et al., 2012; Walker et al., 2009). In these studies, researchers also typically observe hyperalgesia after a subsequent insult (Walker et al., 2009), but others have also reported decreased pain behaviors (Sternberg et al., 2005) in adulthood. Some early-life pain studies report widespread, generalized hypoalgesia to stimulations that occur somewhere other than the site of initial injury (Ren et al., 2004) or in response to more mild pain experiences (Sternberg et al., 2005). Injury recruits an inflammatory neuroimmune response and results in inflammation at the site of injury and the surrounding tissue. As such, researchers also use models of acute or chronic inflammation to study the impacts of neonatal pain and often find that hypersensitivity can be observed into adulthood (Walker, 2013). While there is significant variability across these models (Williams and Lascelles, 2020; Victoria and Murphy, 2016b), a similar conclusion can be drawn from them—adverse painful experiences during early development result in, typically maladaptive, changes to behavioral and physiological responding to a variety of later-life environments and experiences—highlighting the importance of improving our understanding of the repercussions of early-life pain.

Although more widely known for its role in anxiety and fear-based learning (LeDoux, 2000), the amygdala is also a key brain structure involved in pain processing, specifically the emotional and affective aspects of pain (Neugebauer, 2020; Hasanein et al., 2008; Manning et al., 2003; Manning and Mayer, 1995; Helmstetter, 1992; Helmstetter and Bellgowan, 1993; Pomrenze et al., 2019). Changes to amygdala structure (i.e., decreased amygdala volume inversely correlated with the invasive procedures endured) have been observed following time spent in the NICU (Chau et al., 2019), which could impact normal amygdala function and alter responses to future pain and stress. The emotional integration of a painful experience occurs in a subnucleus of the amygdala called the central nucleus of the amygdala (CeA; Neugebauer, 2015). The CeA receives direct nociceptive input from the parabrachial nucleus and projects to other modulatory areas (Neugebauer, 2020; LeDoux, 2000; Chen et al., 2022; Rouwette et al., 2012; Wilson et al., 2019), making it a key target for investigating stress-induced changes to pain perception and fear/anxiety/pain responses. Within the CeA, responses to pain and stress have been shown to vary between normal and pain-state conditions, such that this area has been shown to exhibit both pro- and anti-nociceptive qualities (Wilson et al., 2019). More recently, the role of the central amygdala in pain has also been shown to be lateralized, with the right CeA particularly implicated in pain (Neugebauer, 2015; Chen et al., 2022; Wilson et al., 2019; Carrasquillo and Gereau, 2008; Ji and Neugebauer, 2009; Carrasquillo and Gereau, 2007; Sadler et al., 2017). Indeed, some have found that while the right hemisphere drives pronociceptive properties upon stimulation, the left hemisphere drives antinociceptive properties instead (Ji and Neugebauer, 2009; Sadler et al., 2017; Allen et al., 2023; Kolber et al., 2010). Even within this noted lateralization of pain to the right CeA, researchers observe differences in how neurons of the CeA are impacted. The pro- and anti-nociceptive properties of the CeA may also be due in part to the heterogeneous nature of the neurons in this region, particularly regarding neuropeptide expression (Neugebauer et al., 2020). For example, neurons in the CeA expressing protein kinase C-delta (PKCδ) have been shown to be hyperactive after nerve injury, and chemogenetic manipulation of these neurons modulated pain responses—where inhibition resulted in the reversal of thermal and tactile hypersensitivity after nerve injury, and excitation induced tactile hypersensitivity in the absence of injury—such that it has pro-nociceptive effects (Wilson et al., 2019). Moreover, neurons in the CeA expressing the neuropeptide somatostatin (SOM) have been shown to be altered—where excitation of these neurons reverses hypersensitivity after injury—such that it plays an anti-nociceptive role (Wilson et al., 2019).

Another type of neuron currently being investigated for its role in pain is cells that express corticotropin-releasing factor (CRF, also called corticotropin-releasing hormone, CRH)—a stress hormone that also acts as a neuromodulator in regions like the CeA (Neugebauer et al., 2020; Bale and Vale, 2004; Merchenthaler, 1984; Mazzitelli et al., 2022; Andreoli et al., 2017). Pain-related changes to neurons expressing CRF in the CeA have been assessed in different models (Mazzitelli et al., 2022, 2021) and typically reveal increased activity in pain states. Driving the activity of CeA-CRF neurons under normal conditions results in nocifensive and emotional responses similar to those observed in pain states (Mazzitelli et al., 2021). In a model of neuropathic pain, it was revealed that CRF cells in the CeA are hyperexcitable, specifically during the acute stage of neuropathic pain (Kiritoshi et al., 2024), and play a role in the transition to chronic neuropathic pain. Similar findings have been shown with immediate early gene activation (Butler et al., 2017). Moreover, modulating the activity of CeA-CRF neurons in neuropathic pain impacted emotional-affective responses to pain, with optogenetic silencing resulting in decreased responses at both acute (1 week) and chronic (4 weeks) stages, and optogenetic activation of CeA-CRF neurons in sham controls increasing emotional-affective pain responses when tested 4 weeks post-surgery (Mazzitelli et al., 2022). This study also demonstrated changes to anxiety-related behaviors as a result of manipulating CeA-CRF neurons—with silencing of CRF-expressing neurons in the chronic stage of neuropathic pain having anxiolytic effects and activation in sham controls having anxiogenic effects (Mazzitelli et al., 2022). Studies of early-life pain have also revealed changes to CRF and CeA-CRF neurons after neonatal injury. Specifically, in our lab, early-life pain endured in our model of an NICU-like experience has been shown to acutely increase the amount of CRF in the CeA as well as activation of CRF-expressing cells (Plotsky et al., 2005; Zuke et al., 2019), although we have observed a subsequent, perhaps compensatory, decrease in the CRF cell population of the right CeA as male subjects age (Davis et al., 2021). However, no current work has yet established the necessity of these changes in CRF expression for the lasting effects of neonatal pain observed into adolescence.

This study aims to elucidate the neurobiological changes underlying the maladaptive, pro-pain response to later-life stress observed after early-life injury, with a specific focus on the amygdala and CRF-expressing cells in its central nucleus (CeA-CRF neurons). Our lab employs a two-hit model of juvenile pain vulnerability with repetitive needle prickings during the first week of life (“NICU-like experience”) followed by adolescent fear conditioning (Davis et al., 2018, 2020; Davis and Burman, 2021) and has previously observed a reduction in the number of central amygdala neurons expressing CRF during adolescence. We hypothesize that the remaining population of CeA neurons expressing CRF is functionally altered by our NICU model and primed to respond more readily, such that vulnerability to stress-induced hypersensitivity is increased. To test this, we chemogenetically silenced the central amygdala and occasionally surrounding structures (Experiment 1) or CeA-CRF neurons (Experiment 2) during a subsequent stress event (e.g., fear conditioning) and then assessed mechanical sensitivity. To accomplish the latter study, we used a CRF-Cre rat that expresses Cre-recombinase only in CRF-expressing cells, with particular fidelity in GABAergic cells such as those in the CeA (Pomrenze et al., 2019, 2015). Furthermore, we assessed long-lasting an NICU-like experience-induced changes to the electrophysiological properties of CeA-CRF neurons (Experiment 3) that may be present during exposure to subsequent trauma and alter responding.

## 2 Methods

**Figure 1:**
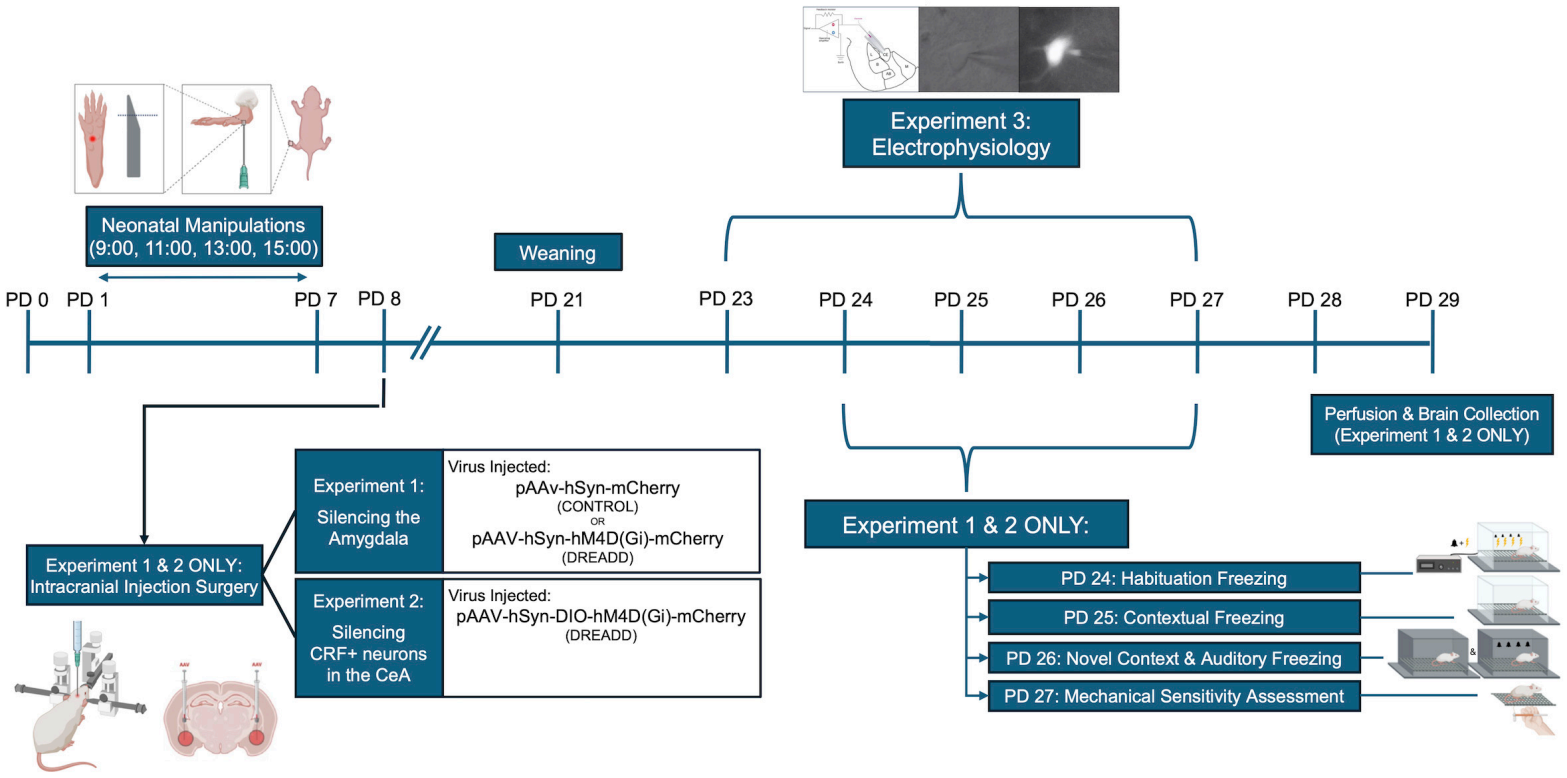
Experimental timeline. Overview of neonatal and juvenile manipulations for subjects in Experiments 1–3. Created in BioRender. Tomasch, M. (2025) https://BioRender.com/zwkssnl.

Subjects underwent both neonatal and juvenile manipulations. A timeline of procedures is presented in Figure 1.

### 2.1 Subjects

For our first chemogenetic experiment, targeting the entire amygdala, a total of 44 male rats were used −24 that did not endure the NICU-like experience [eight non-injected, eight injected with pAAV-hSyn-mCherry, and eight with pAAV-hSyn-hM4D(Gi)-mCherry injected] and 20 that did endure the NICU model [seven non-injected, seven injected with pAAV-hSyn-mCherry, and six with pAAV-hSyn-hM4D(Gi)-mCherry injected]. For our second chemogenetic experiment, targeting the CeA-CRF neurons, a total of 57 male rats were used (36 Cre-negative and 21 Cre-positive)-−32 that did not endure the NICU-like experience [14 non-injected and 18 with pAAV-hSyn-DIO-hM4D(Gi)-mCherry injected] and 25 that did endure our NICU model with pAAV-hSyn-DIO-hM4D(Gi)-mCherry injected.

For our electrophysiology experiment, a total of 30 transgenic male rats were used −16 that did not endure NICU-like experience and 14 that did endure the NICU-like experience. All rats were treated in accordance with the NIH Guide for the Care and Use of Laboratory Animals and approved by the University of New England's Institutional Animal Care and Use Committee (IACUC #011822-002). All rats were housed in 43 × 44 × 20 cm closed-environment cages (Innovive) on a 12-h light/dark cycle, with lights on at 7 a.m., and were provided food and water *ad libitum*.

For our chemogenetic experiment silencing the entire amygdala, we used Sprague Dawley rats (SD; Charles River). For our chemogenetic experiment silencing CeA-CRF neurons, crh-Cre transgenic rats, gifted from Dr. Robert Messing—which express Cre-recombinase in CRF-positive neurons with high fidelity (99%; Pomrenze et al., 2015)—were backcrossed onto a Sprague Dawley background for an average of 17 generations. These rats were bred in-house and were then crossed with Sprague Dawley rats (SD; Charles River), resulting in litters with hemizygous crh-Cre rat pups.

For our electrophysiology experiment, visualization of our neurons of interest was achieved using transgenic rats that have been engineered to express a fluorescent transgene (tdTomato) in our target population, CRF cells. tdTomato reporter knock-in rats [HsdSage: LE-Rosa26em1(tdTomato)Sage] —which express the fluorophore tdTomato behind a floxed stop codon in the Rosa26 locus under control of the CAG promoter that, in the presence of Cre-recombinase, leads to observation of tdTomato fluorescence anywhere Cre is expressed—were purchased from Sage and backcrossed onto a Sprague Dawley background for approximately 20 generations. These rats were then bred with the Crh-Cre transgenic rats. When the tdTomato reporter rats are crossed with the CRF-Cre rats, pups that express Cre-recombinase as well as the tdTomato transgene, with either homo- or heterozygosity, will fluoresce tdTomato specifically in CRF neurons.

Our in-house breeding procedure, explained below, was similar to that of other published works (Davis et al., 2018, 2020; Davis and Burman, 2021; Zuke et al., 2019) and is described here in brief. To synchronize estrus cycles, female rats (PD60+) received subcutaneous injections of deslorelin acetate, a GnRH analog. Eight days later, they were single-housed and paired with a male rat for approximately 24 h. Lab members visually inspected the dams 19 days later, looking for pregnancies. Approximately 50% became pregnant. All rats were born on gestational day 22, which was also postnatal day (PD) 0. Experimental interventions and neonatal manipulations began on PD1.

### 2.2 Neonatal manipulations

On PD 1, pups were removed from their mother, placed on a heating pad, sexed, and culled to no more than 10 rats per litter (five males and five females, when possible). Litters were pseudo-randomly assigned to either neonatal manipulation (“NICU-like”) or undisturbed control condition. Pups in litters designated for neonatal manipulation in our NICU-like condition received a hind paw prick designed to break the dermal layer and draw a drop of blood, in which a 23-gauge needle was inserted into their left hind paw to a depth of approximately halfway up the beveled tip, four times per day, every 2 h (9:00, 11:00, 13:00, 15:00), from PD1 to PD7. While receiving this hind paw prick, the pups were separated from the dam for a minimum of five (and an average of approximately eight) minutes. Animals in the undisturbed condition were left alone, apart from experiment-specific maintenance of animal ID markings (described below). Pups for all experiments were weaned on PD21 into cages with their same-sex littermates (no more than five per cage). All animal procedures were performed in accordance with the University of New England Institutional Animal Care and Use Committee and in compliance with NIH animal care and use guidelines.

In the transgenic rats designated for use in our electrophysiology experiments, a sample of tissue was collected (on PD1) from the rats and sent to the Genotyping Center of America (Waterville, ME) for genetic testing of the TdTomato reporter and Cre-recombinase. To obtain the sample, pups were briefly anesthetized with isoflurane, and 1 mm of cartilaginous tissue from the tip of the tail was cut using a scalpel blade that had been heated in a glass bead sterilizer to immediately cauterize the injury. Rat pups in the NICU-like condition received their first neonatal manipulation prior to anesthetization and tissue sample collection. The pups were marked with crystal violet stain to indicate an identification number, and this staining was redone/updated as needed [an average of once every 3 days for undisturbed, and approximately daily for NICU-like condition due to the additional maternal licking and grooming (Davis et al., 2020)] during PD1–7 before being transitioned to permanent marker tail markings on PD10 (which were redone on PD13, PD18, and PD21).

### 2.3 Chemogenetics

#### 2.3.1 Stereotaxic injections

In the rats designated for use in our chemogenetic experiments, stereotactic bilateral intracranial injections of adeno-associated viral vectors encoding a designer receptor exclusively activated by designer drugs (DREADD) were performed on randomly selected subjects from undisturbed and experimental litters on PD 8. While more recent literature has highlighted different, potentially opposing roles for the left and right amygdala in pain (Ji and Neugebauer, 2009; Sadler et al., 2017; Allen et al., 2023; Kolber et al., 2010), many studies have modulated the activity of both the left and right hemispheres simultaneously (Neugebauer, 2020; Hasanein et al., 2008; Manning et al., 2003; Manning and Mayer, 1995; Helmstetter, 1992; Helmstetter and Bellgowan, 1993; Pomrenze et al., 2019; Neugebauer, 2015). Given this uncertainty, we chose to bilaterally manipulate the amygdala in our experiment. For our experiment targeting all cells in the CeA, we used a pAAV-hSyn-hM4D(Gi)-mCherry (AAV-8) virus. Injection of a non-active virus [pAAV-hSyn-mCherry (AAV-8)] was used to control for the surgery, viral transduction, and expression of the genome. For our experiment targeting CeA-CRF neurons, we injected a Cre-dependent pAAV-hSyn-DIO-hM4D(Gi)-mCherry (AAV-5) to express an inhibitory receptor exclusively in crh-Cre-expressing cells. Given that the virus is only expressed in Cre-expressing rats, animals that did not express Cre (Cre-) were used as injection and viral controls. Tails were labeled using a permanent marker for animal identification, which was reapplied on PD 15 and 21.

The neonates were anesthetized using a 2% concentration of isoflurane (Stoelting, Wood Dale, IL, United States) mixed with 100% oxygen (MaineOxy, ME, United States) in an induction chamber before they were placed in the stereotactic frame (Stoelting, Wood Dale, IL, United States) and fitted with a nose cone. A sagittal incision was made to expose the skull, and the injection site was determined based on rat brain atlas coordinates relative to bregma. We initially used the following coordinates: 1.5 mm caudal to bregma, 4.1 mm lateral to midline, and 6.5 mm below the dura. Over the course of the experiment, to ensure more consistent viral vector expression in the CeA and surrounding structures, these coordinates were shifted slightly to the following: 1.5 mm caudal to bregma, 3.6 mm lateral to midline, and 6.5 mm below the dura, which resulted in consistently greater viral expression in the central and basolateral amygdala. In the experiment targeting the entire amygdala, the first four undisturbed and the first three NICU-like subjects were injected at the initial coordinates, while the last four undisturbed and the last five NICU-like subjects were injected at the final coordinates. The initial coordinates were used for most of the animals in the CeA-CRF experiment, except for the last five in both the undisturbed and NICU-like conditions.

### 2.4 Fear conditioning

On PD 24, the subjects received an intraperitoneal injection of CNO (2 mg/kg) before being transferred to transparent storage boxes where they were housed for 30 min to ensure metabolization and sufficient concentrations of CNO in the amygdala for DREADD activation (Jendryka et al., 2019). Due to the potential for CNO to reverse-metabolize into the atypical antipsychotic clozapine (Manvich et al., 2018), it was necessary to administer CNO to all subjects prior to fear conditioning to account for any behavioral effects associated with clozapine. The subjects were subsequently transferred to Startfear fear conditioning chambers (FCC; Harvard Apparatus/Panlab model #5872) with one of two contextual cues: a square plexiglass chamber with black walls scented with 70% ethanol or a circular plexiglass chamber with white walls scented with 0.5% ammonia.

The fear conditioning protocol started with a 5-min habituation freezing assessment (termed habituation freezing). This was followed by 10 tone-shock pairings, with each pairing starting with a 10-s tone-conditioned stimulus at 67 dB, followed by a 2-s, 1.0 mA foot shock. Following the completion of the 32-min fear conditioning protocol, the subjects were placed back in their respective home cages.

On PD 25, the subjects were placed in the same FCC with the same contextual cues from the previous day, and percent freezing was recorded for 5 min (termed contextual freezing). On the following day (PD 26), the subjects were placed in a different FCC with different contextual cues from the 2 previous days, and percent freezing was recorded for 5 min (termed novel contextual freezing). This was followed by a percent freezing assessment during 10-s, 67 dB tones imitated every 30 s for a total of 10 cycles (termed auditory freezing). To parse out the specific influence of the tone, cued fear, and any fear from being in a novel context, we calculated the difference between these measures (termed tone difference). No CNO was injected during these tests.

### 2.5 Mechanical sensitivity assessment

On PD 27, the tactile withdrawal threshold of each subject was recorded using the up–down von Frey method. The subjects were transferred to plexiglass chambers with mesh floors for a 15-min habituation period. As described in several previously published works (Davis et al., 2021, 2018; Davis and Burman, 2021), von Frey microfilaments (North Coast Medical, Gilroy, CA, United States) of varying thicknesses (0.4 grams to 15 grams of force) were applied to the left hind paw to assess tactile withdrawal threshold. CNO was not injected during this test.

### 2.6 Transcardial perfusion and tissue preservation

On PD 29, the rats were euthanized with a 0.3 mL intraperitoneal injection of sodium pentobarbital (390 mg/mL) and phenytoin sodium (50 mg/mL) before a transcardial perfusion using a 0.9% heparinized saline solution and 4% paraformaldehyde (PFA) was performed. Brains were removed and subsequently post-fixed in 4% PFA for 24 h at 4 °C before being moved to a 30% sucrose solution at 4 °C.

### 2.7 Histology and imaging

After a minimum of 48 h in the 30% sucrose solution, the brains were frozen in optimal cutting temperature (OCT, Tissue-Tek) solution using liquid nitrogen. The frozen brains were then cut into 20-μm slices at −20 °C using a Leica 1950 Cryostat and mounted on charged premium microscope slides (McKesson). The slides were washed with 1X phosphate buffered saline (PBS) and a DAPI mounting media was applied before being covered with #1 coverslips (Sigma-Aldrich). Slides were imaged using a Keyence BZ-X710 fluorescent microscope.

### 2.8 Electrophysiology

#### 2.8.1 Preparation of brain slices

Electrophysiology experiments on the right CeA occurred the first week post-weaning (PD 23–27). TdTomato-positive/crh-Cre-positive male rats, aged P23–P27, underwent live rapid decapitation (with DecapiCone restraint), and their brains were quickly removed. In line with other electrophysiological studies investigating the amygdala's pronociceptive role, which has been shown to be lateralized to the right hemisphere (Wilson et al., 2019; Ji and Neugebauer, 2009; Adke et al., 2021), we focused on the right amygdala (approx. −1.46 mm to −1.58 mm from bregma), and collected 300-μm-thick coronal brain slices using a Leica vibratome in oxygenated, ice-cold high sucrose cutting solution (pH 7.3–7.4) composed of: 206.0 mM sucrose, 2.5 mM KCl, 2.5 mM CaCl_2_, 7 mM MgCl_2_, 1.2 mM NaH_2_PO_4_, 26 mM NaHCO_3_, 5 mM glucose, and 5 mM HEPES. Slices were hemi-sectioned and trimmed and the right hemispheres CeA slices were transferred to a holding chamber filled with artificial cerebral spinal fluid (aCSF) continuously saturated with 95% O_2_/5% CO_2_ and were incubated at 35 °C for 45 min, followed by a minimum 45 min equilibrium to room temperature (21–22 °C). Slices were stored at room temperature for the duration of recordings (1–5 h). The extracellular aCSF was composed of the following (in mM): 125 NaCl, 2.5 KCl, 1.25 NaH_2_PO_4_, 25 NaHCO_3_, 1 MgCl_2_, 2 CaCl_2_, and 1 glucose (pH = 7.2–7.3; osmolarity = 302 mM/kg).

#### 2.8.2 Patch clamp recordings

In TdTomato-positive/crh-Cre-positive rats, TdTomato transgene expression was used for the identification of the CeA and CRF neurons. We visualized neurons using infrared differential interference contrast (IR-DIC) optics and a DAGE-MTI camera. A 40× magnification water immersion objective (Olympus) was used for identifying and approaching neurons with the pipette.

All recordings occurred in a drug-free perfusate at 30–32 °C. Recordings were obtained with borosilicate glass pipettes (4–7 MΩ) fashioned from a programmed pipette puller (P-97 Sutter Instrument Co.) and were filled with a low chloride, potassium-based internal solution composed of the following (in mM): 135 KMeSO_3_, 3 KCl, 1 EGTA(KOH), 10 HEPES, 0.1 CaCl_2_, 4 Mg-ATP, 0.3 Na-GTP, 8 Na_2_-Phosphocreatine, and 0.2% biocytin (pH = 7.2–7.3; osmolarity = 295 mM/kg). A liquid junction potential correction of −8.16 mV was used. Series resistance was monitored (membrane test window) but not compensated for. All neurons were recorded in whole-cell configuration (Molecular Devices Axon DigiData 1550B, Molecular Devices MultiClamp 700B, pCLAMP Clampex Software Version 11), filtered at 3 kHz and digitized at 10 kHz. We began by recording neurons in current-clamp mode for 3–4 min to observe resting membrane potential and possible spontaneous activity. Different series of current injections were used for the assessment of membrane properties. The first series investigated membrane sag potential (*I*_*h*_) with 1-s-long current injections from −200 to 200 pA in 50 pA steps. Next, current injections (500 ms) of increasing amplitude (20 pA steps, from 0 to 400 pA) were delivered to create an input-output curve representing the number of action potentials fired in response to each. To assess input threshold and rheobase, cells were delivered a continuously ramping current injection from 0 to 400 to 0 pA over 30 s. A large depolarizing current injection (700 pA, 500 ms) was used to assess the membrane afterhyperpolarization potential. Researchers determined action potential firing phenotypes visually, defining regular spiking neurons as ones that fire an action potential without delay upon stimulation and late-firing neurons displaying an action potential onset delay of >100 ms (Adke et al., 2021; Martina et al., 1999; Lopez De Armentia and Sah, 2004; Chieng et al., 2006; Li et al., 2022).

pCLAMP Clampex Software (version 11), EasyElectrophysiology, and manual confirmation by the experimenter were used to assess our data. Input–output curves were generated through manual counting of action potentials fired in response to each of the 500 ms current injections (increasing in value from 0 pA to 400 pA in 20 pA steps) in pCLAMP. Rheobase was defined as the current injection value at which the neuron reached action potential firing threshold, and this was determined visually by the experimenter in pCLAMP. Input resistance was calculated in EasyElectrophysiology as the slope of a linear ordinary least squares fit, x = ΔIm, y = ΔVm, for hyperpolarizing current injections from −200 to −50 pA (50 pA steps, 500 ms each). Membrane sag potential was calculated in EasyElectrophysiology as the difference between the steady-state response and the negative peak membrane potential during a 500 ms −200 pA current injection.

### 2.9 Data analysis

#### 2.9.1 Chemogenetics

The data analysis was conducted using the statistical software Prism 10. Two-way ANOVAs with Tukey's *post hoc* analyses were performed when indicated. We treated the litter as the unit of analysis, and the experiment was conducted blind to the genotype. Therefore, when multiple pups from the same litter, genotype, and experimental condition were identified, the data were averaged into a single data point for analysis.

#### 2.9.2 Electrophysiology

All statistical analyses were performed in Prism. A ROUT outlier test (Q = 1%) was run for all measures and datapoints were excluded accordingly (only one cell excluded for sag potential). Descriptive statistics and Kolmogorov–Smirnov normality tests were performed, and all data were subsequently analyzed using Welch's *t*-test. A 2 (Condition: Undisturbed vs. NICU-like) × 21 (Current Injections from 0 to 400 pA in 20 pA steps) ANOVA was performed to analyze input–output curves.

## 3 Results

### 3.1 Chemogenetic silencing of the entire amygdala disrupted auditory freezing and prevented the emergence of conditioning-induced tactile hypersensitivity in NICU subjects

These experiments were designed to test the hypothesis that amygdala activation during fear conditioning is essential for the emergence of a tactile hypersensitivity in NICU-exposed subjects. Neonatal subjects were exposed to the NICU-like conditions or left undisturbed and received an injection aimed at the CeA of control virus (pAAV-hSyn-mCherry), the active inhibitory dread virus [pAAV-hSyn-hM4D(Gi)-mCherry] or no virus at all on PD 8. All subjects received 2 mg/kg CNO prior to fear conditioning.

#### 3.1.1 Fear conditioning

**Figure 2:**
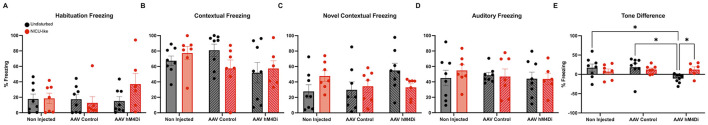
Chemogenetic inhibition of the amygdala during fear conditioning after early-life stress. Percent freezing observed for **(A)** habituation freezing, **(B)** contextual freezing, **(C)** novel contextual freezing, **(D)** auditory freezing, **(E)** tone difference in non-injected, control virus (AAV control) and DREADD (AAV hM4Di) subjects. ^*^p < 0.05. Note that CNO was injected in all subjects only prior to the habituation and subsequent fear conditioning. The remaining tests were conducted without CNO. Black symbols represent the undisturbed group. Red symbols represent the NICU-like condition (all y-axes for **A–D** are percent freezing). X-axes show experimental group.

Percent freezing was assessed following the Pavlovian fear conditioning protocol described above. In line with our previous work (Davis et al., 2018), fear conditioning was only modestly altered by exposure to the NICU-like condition. In addition, there was some evidence that silencing of the amygdala affected fear conditioning (Tallot et al., 2016). There were no significant differences in baseline freezing levels due to NICU-like experience or amygdala silencing prior to the fear conditioning. A 2 (Condition: Undisturbed vs. NICU-like) × 3 [Injection: Non-injected v control virus v hM4D(Gi)] ANOVA revealed no significant main effect of condition *F*_(1, 38)_ = 0.8885, *p* = 0.3519 or injection *F*_(2, 38)_ = 1.057, *p* = 0.3574, or interactions between condition or injection *F*_(2, 38)_ = 1.607, *p* = 0.2137 for habituation freezing (see Figure 2A).

Neither NICU-like experience nor amygdala silencing affected contextual fear conditioning. A 2 (Condition: Undisturbed vs. NICU-like) × 3 [Injection: Non-injected v control virus v hM4D(Gi)] ANOVA revealed there was no significant main effect of condition *F*_(1, 38)_ = 0.1175, *p* = 0.7337 or injection *F*_(2, 38)_ = 1.844, *p* = 0.1721, nor was the interaction between condition and injection significant *F*_(2, 38)_ = 1.785, *p* = 0.1815 for contextual freezing (see Figure 2B).

Both NICU-like experience and amygdala silencing, but not in combination, appeared to slightly increase fear generalization. Although a 2 (Condition: Undisturbed vs. NICU-like) × 3 [Injection: Non-injected v control virus v hM4D(Gi)] ANOVA revealed there was no significant main effect of condition *F*_(1, 39)_ = 0.01521, *p* = 0.9025, or injection *F*_(2, 39)_ = 1.004, *p* = 0.3758, there was a nearly significant interaction between condition and injection *F*_(2, 39)_ = 3.233, *p* = 0.0502. This trending interaction is likely due to different trends in novel contextual freezing after NICU-like experience, where fear generalization was slightly increased in the non-injected group (*p* = 0.0982) and in the hM4D(Gi)-injected group, fear generalization was slightly decreased (*p* = 0.0702; see Figure 2C).

Tone freezing was not impacted by either NICU-like experience or injection status. A 2 (Condition: Undisturbed vs. NICU-like) × 3 [Injection: Non-injected v control virus v hM4D(Gi)] ANOVA revealed there was no significant main effect of condition *F*_(1, 38)_ = 0.1434, *p* = 0.7070 or injection *F*_(2, 38)_ = 0.2732, *p* = 0.7624, and no significant interaction between condition and injection *F*_(2, 38)_ = 0.3097, *p* = 0.7355 (see Figure 2D).

In contrast, amygdala silencing disrupted auditory freezing as assessed by tone difference only in undisturbed subjects. While a 2 (Condition: Undisturbed vs. NICU-like) × 3 [Injection: Non-injected v control virus v hM4D(Gi)] ANOVA revealed no significant main effect of condition *F*_(1, 38)_ = 0.1360, *p* = 0.7143 or injection *F*_(2, 38)_ = 2.060, *p* = 0.1414, there was a trend toward a significant interaction between condition and injection *F*_(2, 38)_ = 2.860, *p* = 0.0696 (see Figure 2E). Subsequent planned comparisons revealed that amygdala silencing successfully disrupted conditioning in undisturbed subjects, as evidenced by reduced tone difference compared to non-injected (*p* = 0.0229) and control-virus injected (*p* = 0.016) subjects, but the same was not true after NICU-like experience, as we detected no significant differences in conditioned freezing between any of the groups for NICU-like subjects. In the active virus injected group (those with their amygdala silenced), the auditory cue resulted in significantly more freezing for those with NICU-like experience (*p* = 0.0405), but no other comparisons were significant. Together, these data suggest that amygdala silencing during fear conditioning disrupted the conditioned fear response to the auditory cue, but only in subjects that did not experience early-life pain. While we anticipated that amygdala silencing would reduce freezing, it was unexpected that early-life trauma would alter the amygdala's role.

#### 3.1.2 Mechanical sensitivity assessment

**Figure 3:**
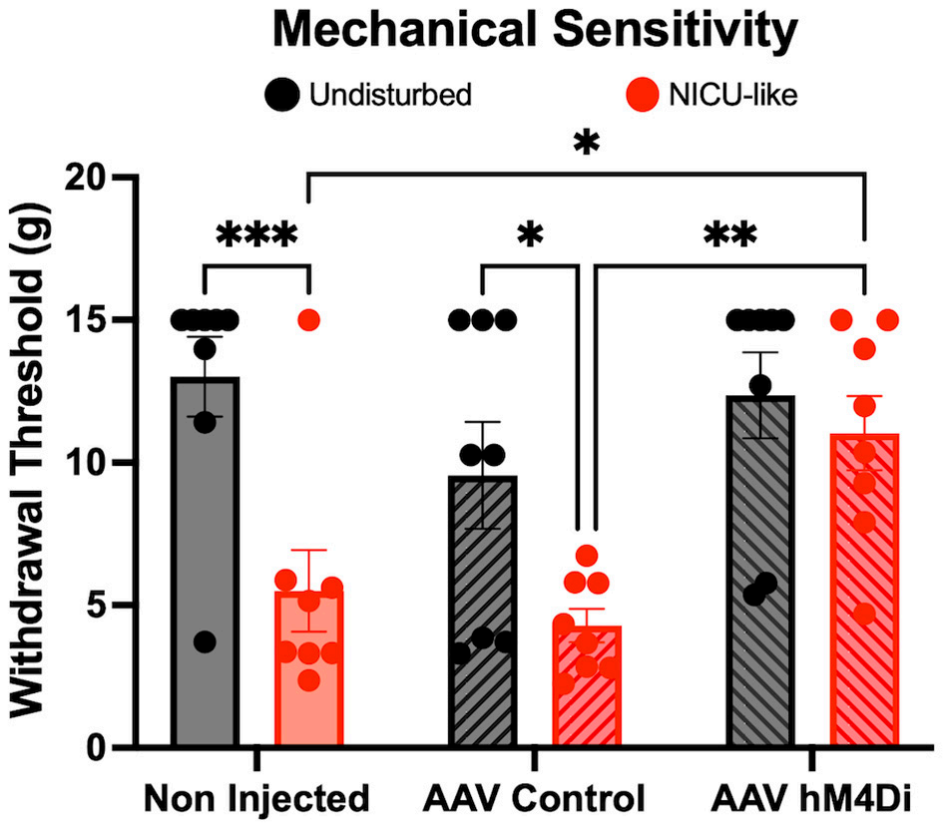
Mechanical sensitivity after chemogenetic inhibition of the amygdala during fear conditioning. Withdrawal threshold (in grams) in response to mechanical stimulation in non-injected, control virus (AAV control) and DREADD (AAV hM4Di) subjects. Note that no CNO was injected prior to this test. Black symbols represent the undisturbed condition. Red symbols represent the NICU-like condition. Significance *p < 0.05, **p < 0.01, ***p < 0.001.

Mechanical sensitivity (See Figure 3) was completely reversed by amygdala silencing during fear conditioning in the NICU-exposed subjects. A 2 (Condition: Undisturbed vs. NICU-like) × 3 [Injection: Non-injected v control virus v hM4D(Gi)] ANOVA revealed a significant main effect of condition *F*_(1, 42)_ = 16.88, *p* = 0.0002 and injection *F*_(2, 42)_ = 5.799, *p* = 0.0060, and a trend toward a significant interaction between condition and injection *F*_(2, 42)_ = 2.492, *p* = 0.0949. NICU-like experience resulted in significantly decreased withdrawal thresholds in both the non-injected (*p* = 0.0005) and control virus-injected (*p* = 0.0110) groups when compared to undisturbed rearing controls. In contrast, hM4D(Gi) group, which had their amygdala silenced, NICU-like experience did not result in decreased withdrawal thresholds (*p* = 0.5067) when compared to the undisturbed rearing controls, suggesting amygdala silencing during fear conditioning blocked development of the conditioning-induced hypersensitivity observed after early-life pain. We also observed that in NICU-like subjects, injection of the active virus was sufficient to significantly increase withdrawal thresholds compared to non-injected (*p* = 0.0213) and control virus-injected (*p* = 0.0041), groups. No other analyses found significant differences.

#### 3.1.3 Analysis of viral expression

**Figure 4:**
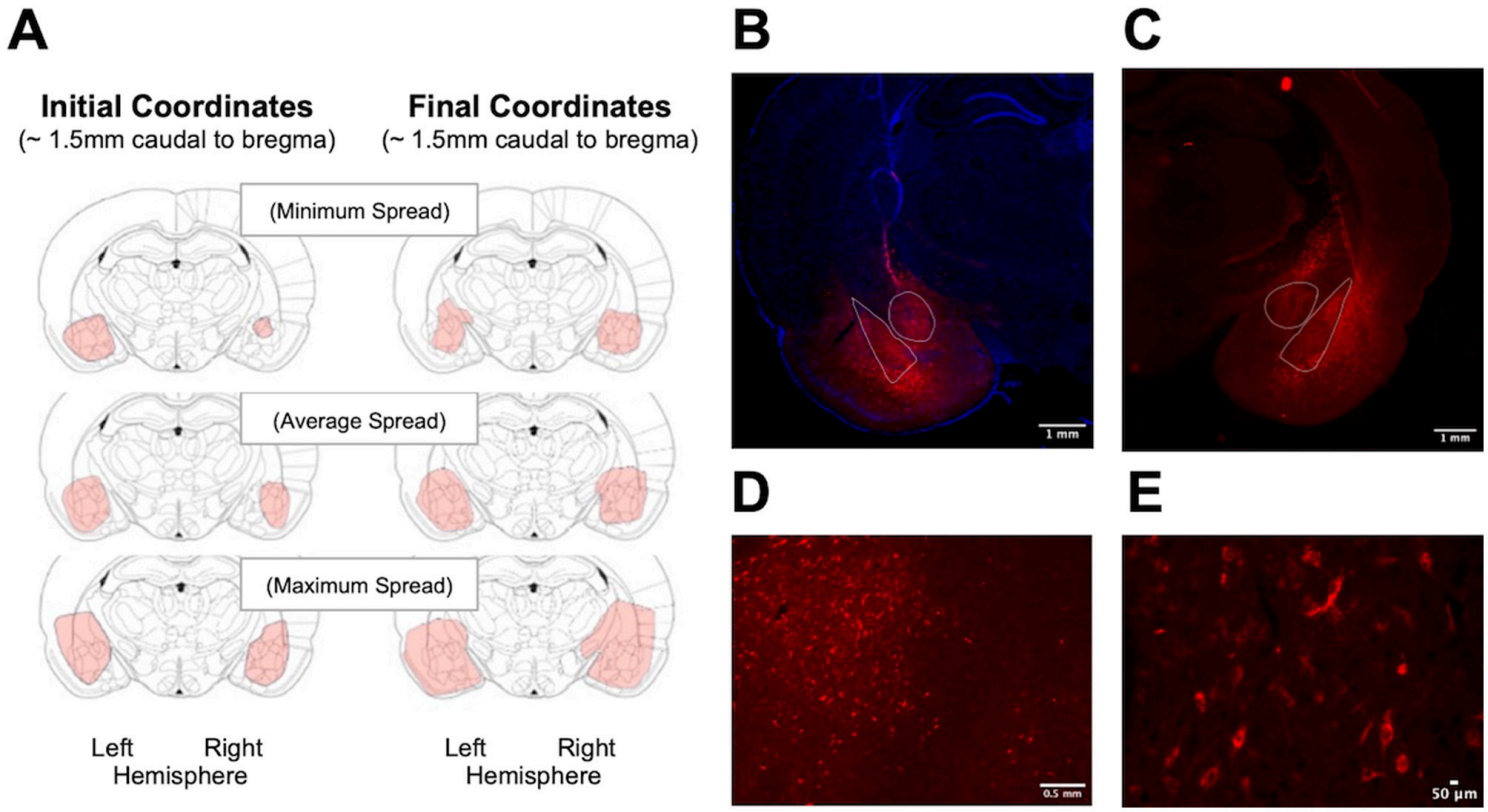
Visualization of pAAV-hSyn-hM4D(Gi)-mCherry expression after bilateral intracranial injections. **(A)** Schematic demonstrating spread of virus [top to bottom: minimum, average, maximum spread of virus injected at (left panel) initial coordinates and (right panel) final coordinates, **(B, C)** two examples of mCherry expression in hemi-sections. **(D)** mCherry expression in the CeA, **E**] high magnification image to show cellular specificity of mCherry expression. Images have been altered for contrast and brightness.

The expression of pAAV-hSyn-hM4D(Gi)-mCherry following bilateral stereotactic injections was assessed using a Keyence BZ-X710 fluorescent microscope. Reconstructions of viral spread were drawn (manually) based on the largest, smallest, and most typical areas of observed mCherry expression (see Figure 4). Expression was observed in all amygdala regions and some surrounding area.

### 3.2 Chemogenetic CeA-CRF neuron silencing only modestly altered auditory freezing and incompletely disrupted the emergence of conditioning-induced tactile hypersensitivity in NICU-exposed subjects

Complete amygdala silencing inhibited fear conditioning and the subsequent tactile hypersensitivity, confirming the role of the amygdala and the efficacy of our DREADD preparation. These experiments were designed to test the hypothesis that CeA-CRF cell activation in particular is essential during fear conditioning for the emergence of a tactile hypersensitivity in NICU-exposed subjects. CRF-Cre+ and CRF-Cre- neonatal subjects were exposed to the NICU conditions or left undisturbed and received an injection aimed at the CeA of the Cre-dependent inhibitory dread virus [pAAV-hSyn-DIO-hM4D(Gi)-mCherry] or no virus at all on PD 8. All subjects received 2 mg/kg CNO prior to fear conditioning.

#### 3.2.1 Fear conditioning

**Figure 5:**
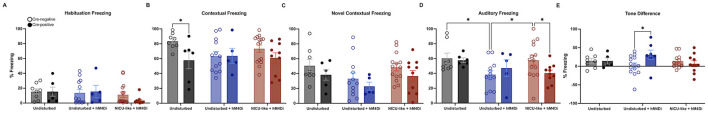
Chemogenetic inhibition of the CeA-CRF neurons during fear conditioning after early-life stress. Percent freezing for **(A)** habituation freezing, **(B)** contextual freezing, **(C)** novel contextual freezing, **(D)** auditory freezing, **(E)** tone difference in control (undisturbed rearing), virus only (undisturbed + hM4Di), and NICU + hM4Di subjects. Note that CNO was injected to all subjects only prior to the habituation and subsequent fear conditioning. The remaining tests were conducted without CNO. Black symbols represent the undisturbed condition. Open circles and lighter bars represent Cre-negative animals. Closed circles and darker bars represent Cre-positive animals. Significance *p < 0.05 (all y-axes for **A–D** are Percent Freezing). X-axes show experimental group.

There were no differences in baseline freezing due to Cre-status or injection group, as a 2 (Cre Status: Cre-negative v Cre-positive) × 3 [Group: Undisturbed vs. Undisturbed with hM4D(Gi) injection v NICU-like with hM4D(Gi) injection] ANOVA revealed no significant main effect of injection group *F*_(2, 51)_ = 2.076, *p* = 0.1360 or Cre-status *F*_(1, 51)_ = 0.2779, *p* = 0.6003, and no significant interaction between injection group and Cre-status *F*_(2, 51)_ = 0.7007, *p* = 0.5010 (see Figure 5A).

It appears that both Cre-status and injection group increase variability in contextual freezing levels, but the effects were not consistent across subjects. A 2 (Cre Status: Cre-negative v Cre-positive) × 3 [Group: Undisturbed vs. Undisturbed with hM4D(Gi) injection v NICU-like with hM4D(Gi) injection] ANOVA revealed a significant main effect of Cre-status *F*_(1, 51)_ = 4.672, *p* = 0.0354, with Cre-positive rats showing reduced contextual freezing that was likely caused by a significant difference between Cre-positive and Cre-negative subjects only in the undisturbed non-injected animals (*p* = 0.0238), as there were no significant effects of Cre status in either virus-injected group. There was no significant effect of group *F*_(2, 51)_ = 0.4212, *p* = 0.6585, and no interaction between Cre-status and group *F*_(2, 51)_ = 1.4626, *p* = 0.2498. In the Cre-negative animals, in which CRF cells were not silenced, there was a trend toward a significant reduction in contextual freezing for undisturbed with hM4D(Gi) injection compared to undisturbed non-injected (*p* = 0.0832), but no other significant differences were detected (see Figure 5B).

Neither Cre-status nor injection group appear to significantly impact novel contextual freezing. A 2 (Cre Status: Cre-negative v Cre-positive) × 3 [Group: Undisturbed vs. Undisturbed with hM4D(Gi) v NICU-like with hM4D(Gi) injection] ANOVA revealed a trend toward a main effect of Cre-status *F*_(1, 49)_ = 3.375, *p* = 0.0723 with Cre-positive animals being lower, and group *F*_(2, 49)_ = 2.559, *p* = 0.0877 with undisturbed-injected being lower, but no interaction between Cre-status and group *F*_(2, 49)_ = 0.009036, *p* = 0.9910. However, there were no significant *post-hoc* pairwise comparisons (see Figure 5C).

In rats that endured the NICU-like experience, silencing of CRF cells in the CeA reduced auditory freezing behavior, but Cre-status, and thus whether CRF cells are silenced, did not significantly impact conditioned freezing in the other groups. A 2 (Cre Status: Cre-negative v Cre-positive) × 3 [Group: Undisturbed vs. Undisturbed with pAAV-hSyn-DIO-hM4D(Gi)-mCherry injection v NICU-like with pAAV-hSyn-DIO-hM4D(Gi)-mCherry injection] ANOVA revealed a significant main effect of group *F*_(2, 49)_ = 2.479, *p* = 0.0043 but no significant main effect of Cre-status *F*_(1, 49)_ = 0.4566, *p* = 0.5024 or interaction between Cre-status and group *F*_(2, 49)_ = 2.087, *p* = 0.1349 (see Figure 5D). In Cre-negative animals, in which CRF cells were not silenced, there was a significant reduction in auditory freezing in undisturbed animals that had control AAV injections when compared to the non-injected undisturbed animals (*p* = 0.0436) as well as the NICU-like with hM4D(Gi) injection group (*p* = 0.0374), suggesting auditory freezing behavior may be reduced after the neonatal intervention of the viral injection surgery alone, but was rescued by the repeated intervention of our NICU model. In the NICU-like with hM4D(Gi) injection group, Cre-positive animals showed significantly less auditory freezing than Cre-negative animals (*p* = 0.0374), suggesting our silencing of CRF cells reduced conditioned fear behavior. Cre status did not significantly impact either group.

Tone difference score appears to be altered in undisturbed animals that had AAV injections. This is likely explained by the changes observed in novel contextual freezing (albeit non-significant differences) and auditory freezing, as a 2 (Cre Status: Cre-negative v Cre-positive) × 3 [Group: Undisturbed vs. Undisturbed with hM4D(Gi) injection v NICU-like with hM4D(Gi) injection] ANOVA revealed only a trend toward a significant interaction between Cre-status and group *F*_(2, 50)_ = 2.905, *p* = 0.0640, with Cre-status significantly impacting tone difference specifically in the undisturbed animals with AAV injections. No significant main effects of Cre-status *F*_(1, 50)_ = 1.059, *p* = 0.3083 or group *F*_(2, 50)_ = 0.3243, *p* = 0.7245, were observed for cued fear response (see Figure 5E). In undisturbed animals that received AAV injections, CRF cell silencing resulted in a significantly increased conditioned fear response, when assessed by tone-difference score, to the auditory cue (*p* = 0.0141).

#### 3.2.2 Mechanical sensitivity assessment

**Figure 6:**
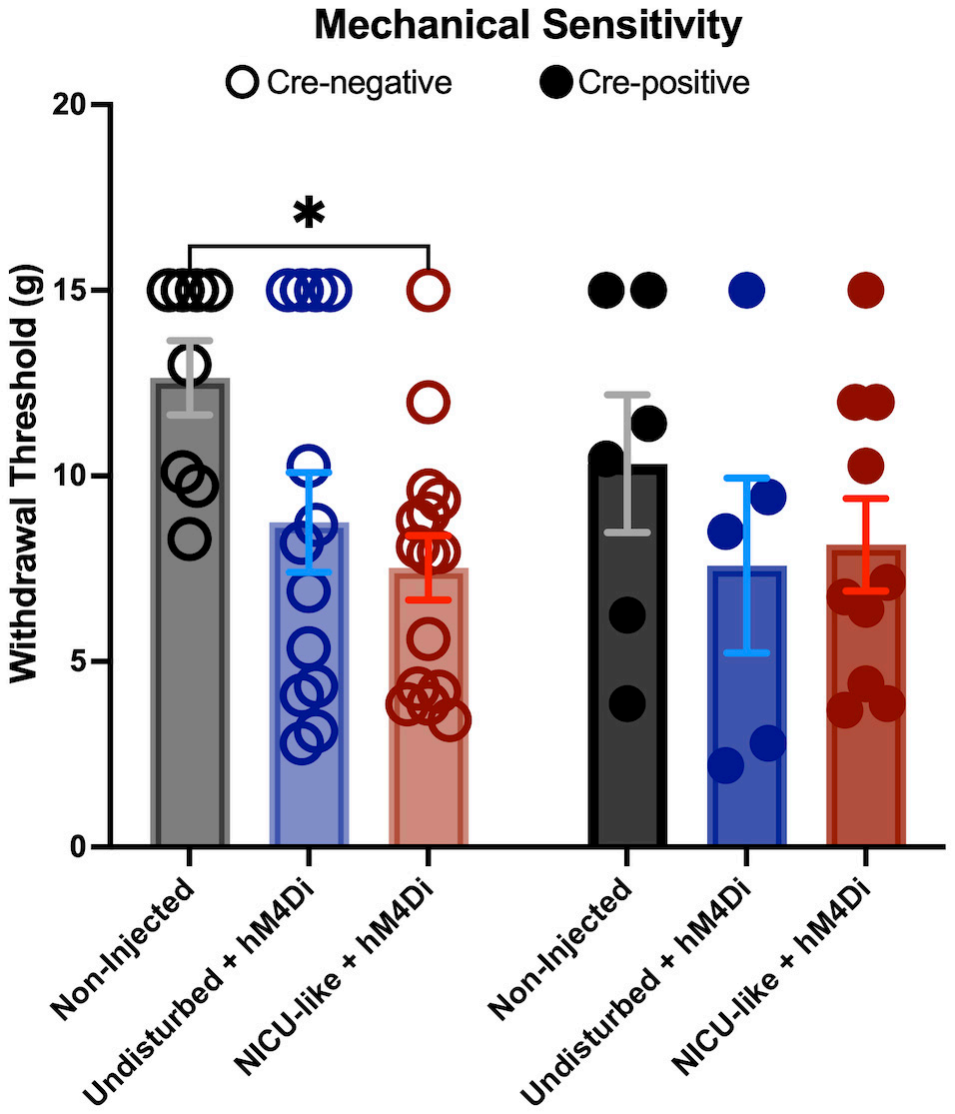
Mechanical sensitivity after chemogenetic inhibition of CeA-CRF neurons during fear conditioning. Withdrawal threshold (in grams) in response to mechanical stimulation in control (undisturbed rearing), virus only (undisturbed + hM4Di), and NICU + hM4Di subjects. Open circles and lighter bars represent Cre-negative animals. Closed circles and darker bars represent Cre-positive animals. Note that CNO was not injected prior to this test. Significance *p < 0.05. X-axes show experimental group.

As expected, NICU-like experience significantly increased mechanical hypersensitivity after later-life trauma (Figure 6). A 2 (Cre Status: Cre-negative v Cre-positive) × 3 [Group: Undisturbed vs. Undisturbed with hM4D(Gi) injection v NICU-like with hM4D(Gi) injection] ANOVA revealed a significant main effect of group *F*_(2, 51)_ = 3.801, *p* = 0.0289, but no significant main effect of Cre-status *F*_(1, 51)_ = 0.6661, *p* = 0.4182 or interaction between Cre-status and group *F*_(2, 51)_ = 0.6039, *p* = 0.5505. In Cre-negative animals, in which the CRF cells were not silenced, there was a significant reduction in withdrawal threshold in subjects from the NICU-like condition (*p* = 0.0164), as we have previously observed. It appears viral injection surgery alone during the neonatal period, without the week of repetitive needle prickings, may have resulted in a slightly increased sensitivity to later stress-induced pain, as there was a trend toward reduced withdrawal threshold in the undisturbed-injected group compared to the undisturbed group that was not injected (*p* = 0.0955). When CRF cells were silenced in the Cre-positive injection groups, there were no significant differences in withdrawal thresholds compared to the non-injected undisturbed subjects (undisturbed with AAV injection *p* = 0.5121; NICU-like with AAV injection *p* = 0.5577). Furthermore, it appears silencing CeA CRF cells blocked the trend observed in Cre-negative subjects where even injection surgery alone induced some mechanical sensitivity after fear conditioning compared to the non-injected group (*p* = 0.9660). However, contrary to our hypothesis that silencing CeA CRF cells during fear conditioning should block or reverse the observed hypersensitivity in NICU subjects, there were no significant pairwise comparisons between Cre-negative and Cre-positive subjects. While this was anticipated in the undisturbed group (*p* = 0.2992) and the undisturbed with hM4D(Gi) injection group (*p* = 0.5898), we had anticipated a significant reversal of hypersensitivity in the NICU-like with hM4D(Gi) injection group that failed to manifest (*p* = 0.7109), raising into question whether CRF cell silencing alone is enough to fully reverse or block trauma-induced hypersensitivity after our NICU model.

#### 3.2.3 Analysis of viral expression

**Figure 7:**
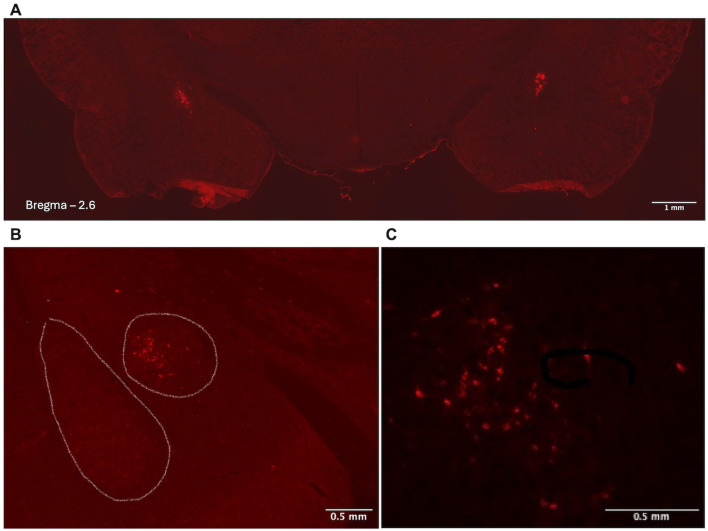
Visualization of mCherry expression in CeA-CRF neurons after bilateral intracranial injections of pAAV-hSyn-DIO-hM4D(Gi)-mCherry. **(A)** Bilateral expression of mCherry is limited to the CeA in a hemi-section. **(B, C)** Higher magnification images to show specificity of labeling. Images have been altered for contrast and brightness.

The expression of pAAV-hSyn-hM4D(Gi)-mCherry following bilateral stereotactic injections was assessed using a Keyence BZ-X710 fluorescent microscope (Figure 7). Note that between 30 and 50 CRF-positive neurons, with an average of 38 cells per section, were observed to express mCherry. This amount of expression is in line with our previous observation using *in situ* hybridization, identifying between 6 and 80 *crh* (mean of 46.8) cells expressed in the right CeA of PD 24 males (Davis et al., 2021), suggesting we successfully transfected the majority of CeA-CRF neurons.

### 3.3 NICU-like experience increases the excitability of CRF neurons in the right CeA

Silencing CeA-CRF cells only modestly impacted conditioning-induced hypersensitivity in our NICU subjects, raising questions about their necessity. This experiment further investigates whether early-life pain and stress alter the physiology of these cells, leaving open the possibility that they are involved in the lasting behavioral changes we observe. To test the impacts of the NICU-like experience on CeA-CRF cells, whole-cell current clamp recordings in acute slices were obtained. This investigation revealed an increase in the excitability of CeA-CRF neurons after NICU-like experience.

#### 3.3.1 Electrophysiological properties of CeA-CRF neurons

**Figure 8:**
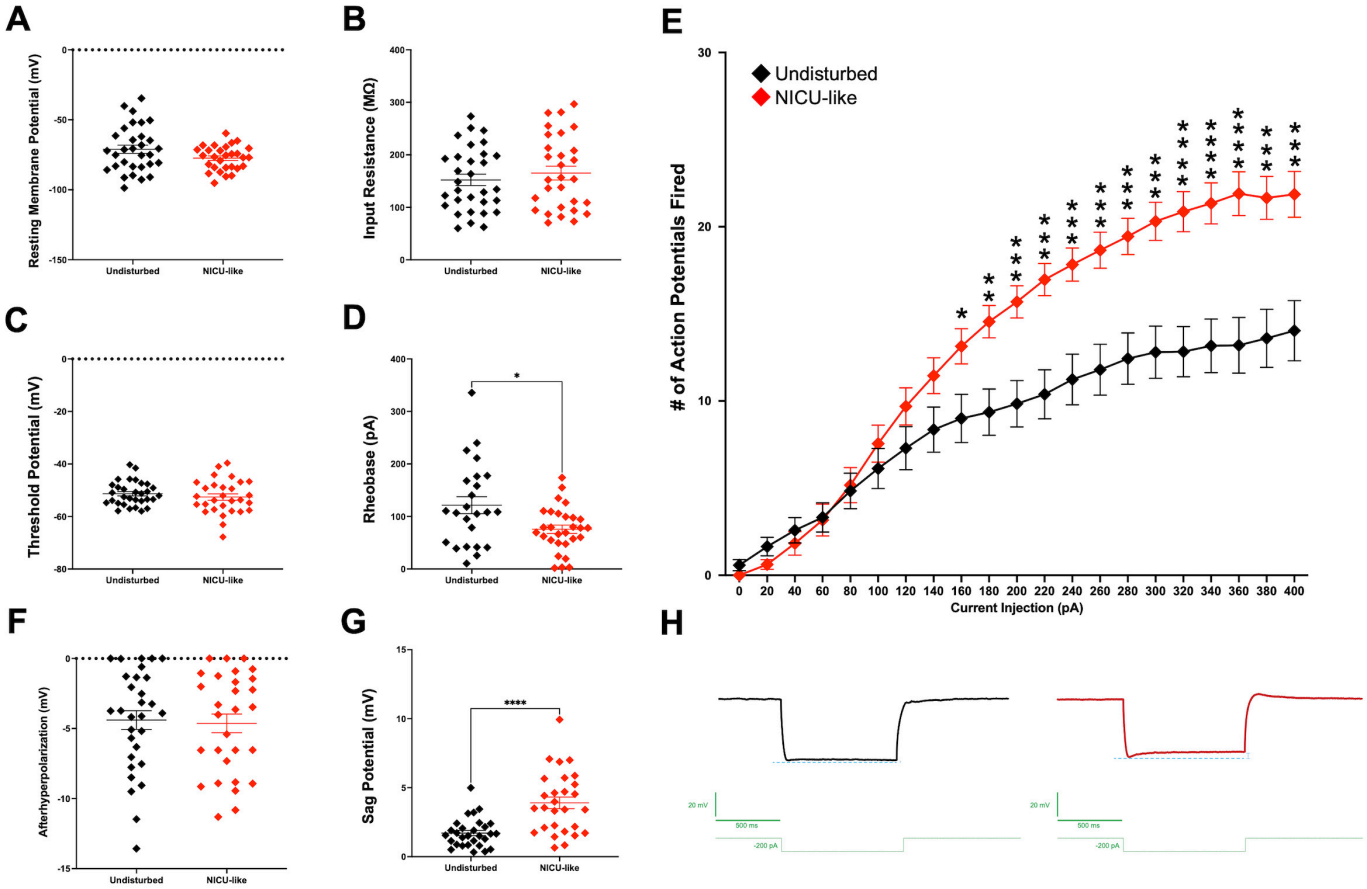
Membrane properties and excitability of CeA-CRF neurons. **(A)** Resting membrane potential (mV), **(B)** input resistance (MΩ), **(C)** firing threshold potential (mV), **(D)** rheobase (pA), **(E)** input-output curve showing number of action potentials fired per current step. **(F)** Afterhyperpolarization potential (mV), **(G)** sag potential (mV), **(H)** representative traces of sag potentials. Black symbols represent undisturbed animals. Red symbols represent NICU-like animals. Significance *p < 0.05, **p < 0.01, ***p < 0.001, ****p < 0.0001.

After exposure to our NICU model, CeA-CRF neurons are hyperexcitable. Although there were no significant differences in resting membrane potential (Figure 8A), input resistance (Figure 8B), or threshold potential (Figure 8C), there was a significant reduction in rheobase, where animals from the NICU-like condition required less current stimulation to fire action potentials (Figure 8D), as revealed by an unpaired *t*-test with Welch's correction *t*_(33.80)_ = 2.542, *p* = 0.0158. Furthermore, in response to current stimulations of equivalent value, cells from animals that had endured the NICU-like experience fired significantly more action potentials (see Figure 8E). A 2 (condition: undisturbed vs. NICU-like) × 21 (current injection: 20 pA steps from 0 to 400 pA) way ANOVA with Tukey's correction revealed significant main effects of both current injection values *F*_(1.915, 110.2)_ = 101.6, *p* < 0.0001 and condition *F*_(1, 58)_ = 14.77, *p* = 0.0003, as well as a significant interaction between current injection and condition *F*_(20, 1151)_ = 8.532, *p* < 0.0001. CeA-CRF cells from NICU-like animals began firing significantly more action potentials in response to a current injection of 160 pA (*p* = 0.0190), and this persisted for the remainder of the injection values (i.e., 180 pA: *p* = 0.0023, 200 pA: *p* = 0.0007, 300 pA: *p* = 0.0002, 400 pA: *p* = 0.0007). No differences in afterhyperpolarization potential were detected after NICU-like experience (see Figure 8F). In the NICU rats, CeA-CRF neurons display stronger I_h_ currents. There is an increased ability to depolarize the membrane in response to hyperpolarization after the early-life pain and stress, such that in response to a −200 pA current injection, there was a significantly increased sag potential in CeA-CRF neurons from animals in the NICU-like condition, as revealed by an unpaired *t*-test with Welch's correction *t*_(38.05)_ = 4.705, *p* < 0.0001 (see Figure 8G).

**Figure 9:**
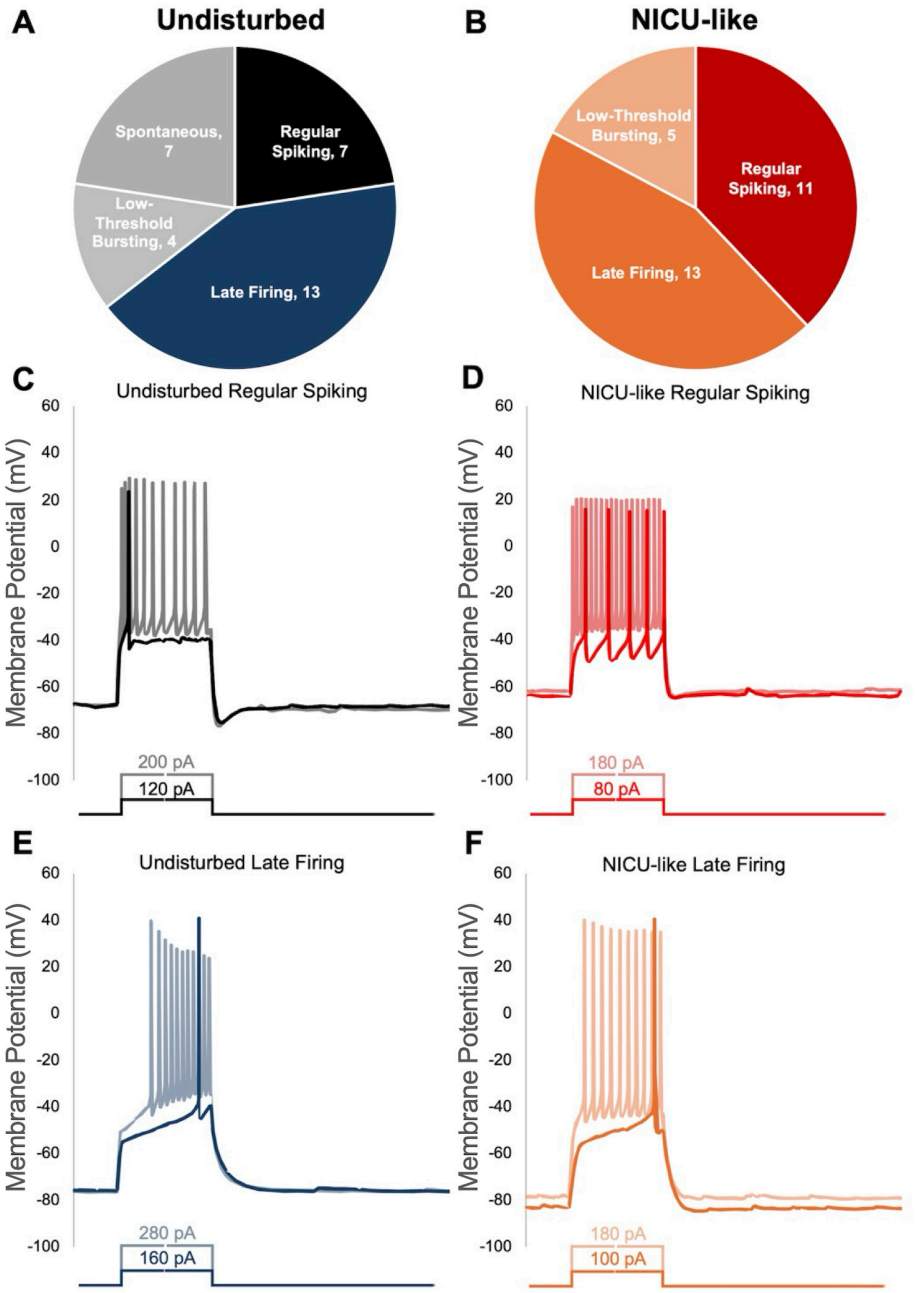
Distribution of action potential firing phenotypes observed in CeA-CRF neurons. **(A)** Breakdown indicating how many of each phenotype were detected in undisturbed subjects, **(B)** breakdown indicating how many of each phenotype were detected in NICU-like subjects, **(C)** representative traces for regular spiking neurons in the undisturbed condition showing the first action potential fired (black) and how the neuron fires at higher stimulation values (gray), **(D)** representative traces for regular spiking neurons in the NICU-like condition showing the first action potential fired (red) and how the neuron fires at higher stimulation values (light red/pink), **(E)** representative traces for late-firing neurons in the undisturbed condition showing the first action potential fired (dark blue) and how the neuron fires at higher stimulation values (lighter blue), **(F)** representative traces for late-firing neurons in the NICU-like condition showing the first action potential fired (orange) and how the neuron fires at higher stimulation values (light orange).

We observed that CeA-CRF neurons display a variety of action potential firing pattern phenotypes (see Figure 9), including regular spiking (RS), late firing (LF), low threshold bursting (LTB), and spontaneously active (S). As there were no spontaneously active cells observed in the NICU-like condition, and low-threshold bursting cells are thought to be a relatively rare CRF cell type in the CeA, we focused our phenotypic analyses on regular spiking and late-firing neurons, which is also reflective of other published literature investigating the impacts of pain on CeA-CRF neurons (see Wilson et al., 2019; Kiritoshi et al., 2024; Adke et al., 2021).

#### 3.3.2 Electrophysiological properties of regular spiking and late firing CeA-CRF neurons

**Figure 10:**
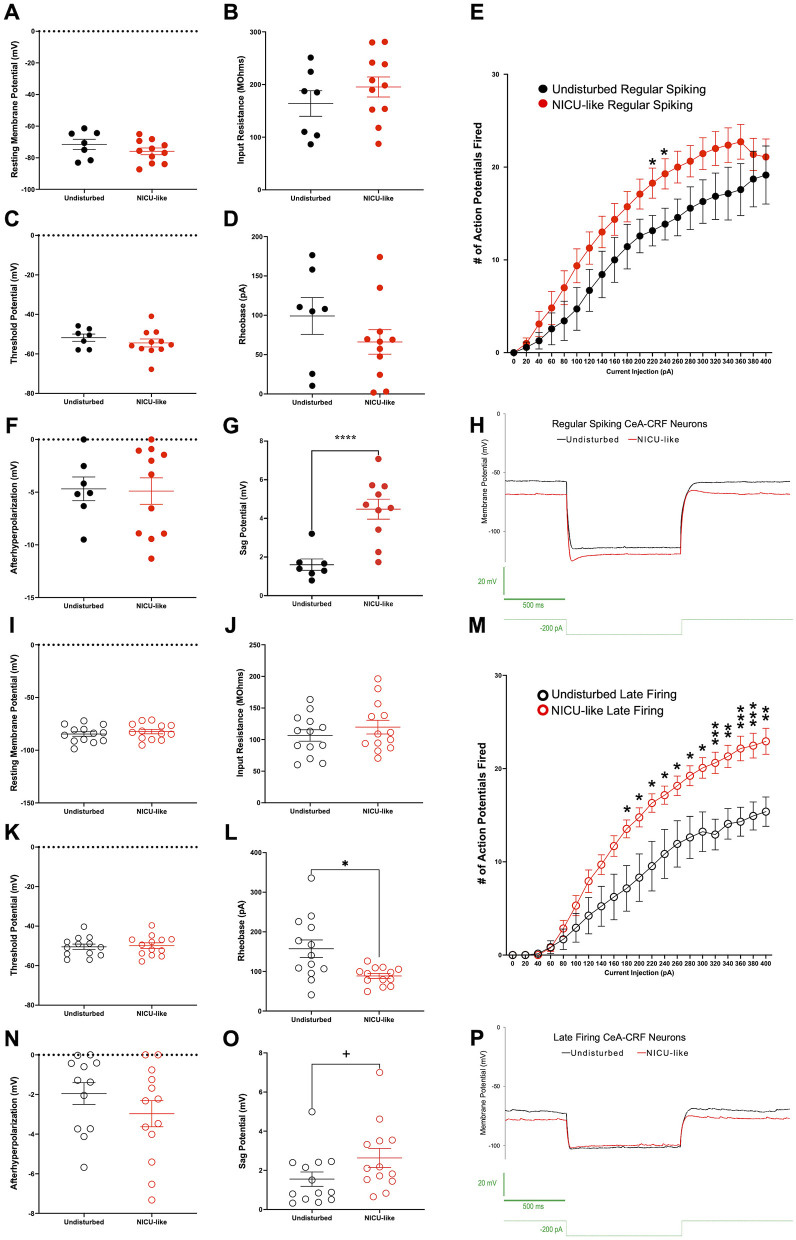
Membrane properties and excitability of CeA-CRF neurons. **(A–H)** Regular spiking CeA-CRF neurons: **(A)** Resting membrane potential (mV), **(B)** input resistance (MΩ), **(C)** firing threshold potential (mV), **(D)** rheobase (pA), **(E)** input–output curve showing number of action potentials fired per current step, **(F)** afterhyperpolarization potential (mV), **(G)** sag potential (mV), **(H)** representative sag potential traces. **(I–P)** Late-firing CeA-CRF neurons: **(I)** resting membrane potential (mV), **(J)** input resistance (MΩ), **(K)** firing threshold potential (mV), **(L)** rheobase (pA), **(M)** input–output curve showing number of action potentials fired per current step, **(N)** afterhyperpolarization potential (mV), **(O)** sag potential (mV), **(P)** representative sag potential traces. Black symbols represent undisturbed animals. Red symbols represent NICU-like animals. Significance ^+^*p* < 0.10, *p < 0.05, **p < 0.01, ***p < 0.001, ****p < 0.0001.

The membrane properties of regular spiking CeA-CRF neurons appear to be unchanged after the NICU-like experience, as we detected no significant changes to regular spiking CeA-CRF membrane properties afterward. Unpaired *t*-tests with Welch's correction did not reveal significant differences in resting membrane potential *t*_(11.06)_ = 1.128, *p* = 0.2831 (Figure 10A), input resistance *t*_(12.76)_ = 1.1016, *p* = 0.3286 (Figure 10B), threshold potential *t*_(15.65)_ = 0.9769, *p* = 0.3435 (Figure 10C), or rheobase *t*_(11.21)_ = 1.173, *p* = 0.2650 (Figure 10D). The excitability of regular spiking CeA-CRF neurons appears to be mostly unchanged after exposure to early-life pain and stress, as we detected only a modest increase in the number of action potentials fired per current step compared to controls after our NICU-like experience (Figure 10E). A 2 (condition: undisturbed vs. NICU-like) × 21 (current injection: 20 pA steps from 0 to 400 pA) way ANOVA with Tukey's correction revealed a main effect of current injection *F*_(1.880, 30.08)_ = 58.06, *p* < 0.0001 and a trend toward a main effect of condition *F*_(1, 16)_ = 3.623, *p* = 0.0751, but no significant interaction between current and condition *F*_(20, 320)_ = 0.8318, *p* = 0.6744. There was a trend toward a significant increase in action potentials fired after NICU-like experience at a current injection value of 200 pA (*p* = 0.0836). NICU-like regular spiking CeA-CRF neurons fired significantly more action potentials than those from undisturbed animals only at current injection values of 220 pA (*p* = 0.0421) and 240 pA (*p* = 0.0372), but the significance of this effect diminished as current injection values increased beyond that (260 pA: *p* = 0.0570, 280 pA: *p* = 0.1009, 300 pA: *p* = 0.1008, 320 pA: *p* = 0.1228, and so on). Furthermore, while no difference in afterhyperpolarization potentials was detected after NICU-like experience (see Figure 10F), there was a significant increase in sag potential (*p* < 0.0001, see Figures 10G, H).

The NICU-like experience does appear to alter the membrane properties of late-firing CeA-CRF neurons and lead to hyperexcitability. Unpaired *t*-tests with Welch's correction revealed no significant differences in resting membrane potential, *t*_(23.95)_ = 0.7658, *p* = 0.4513 (Figure 10I), input resistance, *t*_(23.28)_ = 0.9337, *p* = 0.3601 (Figure 10J), or threshold potential, *t*_(23.97)_ = 0.3047, *p* = 0.7632 (Figure 10K), but there was a significant reduction in the rheobase of late-firing CeA-CRF neurons after exposure to our model, *t*_(13.90)_ = 2.977, *p* = 0.0101 (Figure 10L), demonstrating that these cells are hyperexcitable. Hyperexcitability of late-firing CeA-CRF neurons after NICU-like experience is further demonstrated through an increase in action potentials fired in response to the same current stimulations compared to those from the undisturbed condition. A 2 (condition: undisturbed vs. NICU-like) × 21 (current injection: 20 pA steps from 0 to 400 pA) way ANOVA with Tukey's correction revealed a main effect of current *F*_(2.127, 51.05)_ = 94.89, *p* < 0.0001, and condition, *F*_(1, 24)_ = 8.863, *p* = 0.0066, as well as a significant interaction between current and condition, *F*_(20, 480)_ = 4.311, *p* < 0.0001. A trend toward a significant difference in the number of action potentials fired was observed starting at a current injection value of 140 pA (*p* = 0.0784). A significant difference in the number of action potentials fired was revealed starting at 180 pA (*p* = 0.0277) and continued for the remainder of the injections (Figure 10M). Again, no significant change to afterhyperpolarization potential was observed (*p* = 0.2466, see Figure 10N), and this time there was a trend toward increased sag potential after NICU-like experience (*p* = 0.0887, see Figures 10O, P).

## 4 Discussion

For a summary of results, see Table 1. This study investigated the neurobiological mechanisms underlying lasting changes in fear conditioning behavior and pain perception in a rat model of neonatal medical trauma. Consistent with prior work (Davis et al., 2018, 2020; Davis and Burman, 2021; Zuke et al., 2019), this study suggests neonatal trauma leads to only modest alterations in fear behavior, primarily in the form of fear generalization to novel contexts. Furthermore, we replicate our consistent finding that repeated neonatal injury, followed by adolescent fear conditioning, produces a stress-induced mechanical hypersensitivity (Davis et al., 2018; Davis and Burman, 2021). This is consistent with other published literature indicating that neonatal adversity results in altered pain perception that spans into adulthood. These experiments contributes to the literature by demonstrating that the involvement of the amygdala is critical for the development of the stress-induced hypersensitivity observed in animals that experienced early-life injury and that CeA-CRF neurons may also play a role. This study further demonstrates that early-life adversity, such as that endured in an NICU-like experience, results in hyperexcitability of CeA-CRF neurons, as observed in the acute stages of neuropathic pain (Kiritoshi et al., 2024) and arthritis pain (Mazzitelli et al., 2021). Together, these results suggest that neonatal trauma can profoundly alter the neurobiology of both pain and fear, leading to lasting changes in sensory processing and emotional regulation.

**Table 1:** Summary of behavioral results from the DREADD experiments.

**Experiment**	**Contextual fear conditioning**	**Auditory fear conditioning**	**Hypersensitivity**
Whole amygdala silencing	Not affected Figure 2B; AAV hM4Di (Undisturbed vs. NICU-like)	Disrupted Figure 2E; AAV hM4Di (Undisturbed vs. NICU-like)	Blocked Figure 3; AAV hM4Di (Undisturbed vs. NICU-like)
CeA-CRF+ silencing	Not affected Figure 5B; Cre+ (Undisturbed + AAV vs. NICU-like + AAV)	Disrupted only in NICU-like subjects Figure 5D; Cre+ (Undisturbed + AAV vs. NICU-like + AAV)	Mixed results Figure 6; Cre+ (Undisturbed + AAV vs. NICU-like + AAV)

Our rodent model, designed to replicate some NICU conditions, consistently demonstrates that experimenter handling, brief maternal separation, and repetitive skin-breaking procedures endured during the first week of life create a long-lasting vulnerability to fear conditioning-induced mechanical hypersensitivity (Davis et al., 2018; Davis and Burman, 2021). Studies of procedural pain in neonates, such as repetitive needle prickings and surgical incisions, often reveal that exposure to these painful experiences during the early developmental period increases maladaptive responding to pain and stress in later life, with neonatal pain-exposed animals demonstrating heightened pain responding, increased anxiety-like behaviors, and increased preference for alcohol (Anand et al., 1999; De Carvalho et al., 2019). This is similar to observed outcomes in school-aged children who spent time in the NICU as infants (Grunau et al., 2006), who also demonstrate long-term increased susceptibility to chronic pain, anxiety and depression, and substance use disorders (Brummelte et al., 2015). Furthermore, alterations to the normal function of the amygdala during acutely stressful experiences may impact fear responding (LeDoux, 2000; Tallot et al., 2016; Davis, 1990; LeDoux et al., 1990; Maren and Fanselow, 1996; Ponnusamy et al., 2007; Wilensky et al., 2006), and as such, we expected chemogenetic silencing of the amygdala to impede the acquisition of conditioned fear. We did not detect significant differences in percent freezing between experimental groups for habituation or contextual freezing, but we did observe a trend for contextual freezing resulting from chemogenetic silencing overall, as well as a statistically significant reduction in the tone difference score in control subjects and a reduction in auditory freezing in CRF-silenced NICU subjects. Fear conditioning is an evolutionarily necessary and complex task with a broad circuit (Chaaya et al., 2018); thus, it is possible that other structures can compensate for the lack of amygdala function (Ponnusamy et al., 2007), and this may be especially true in developing animals.

Development of hypersensitivity that occurs specifically after a second activating event or injury is consistent with previously described “two-hit” models of early-life adversity, which posit that both neonatal trauma and a secondary stressor are required for the development of adverse outcomes, such as mechanical hypersensitivity (Williams and Lascelles, 2020; Davis et al., 2018). According to this model, early-life trauma may prime the brain for an abnormal, pronociceptive response to subsequent pain and stress, thereby enhancing susceptibility to pain-related disorders. Nociceptive priming and critical periods of nociceptive development have been assessed both clinically and preclinically (Fitzgerald, 2005; Schwaller and Fitzgerald, 2014; Lidow, 2002; Fitzgerald and Beggs, 2001). Importantly, in preclinical models of neonatal injury, long-lasting hypersensitivity was not observed if the initial injury did not occur within the first 10 days of life (Walker et al., 2009). Furthermore, there is a positive correlation between the number of painful procedures experienced and the degree of hypersensitivity observed (Williams and Lascelles, 2020). This may explain the results observed in our undisturbed but virally injected animals, as they endured a single pain experience during this critical window and displayed a slight hypersensitivity compared to the more pronounced hypersensitivity observed in animals that endured more painful experiences. Together, these results support the conclusion that our model of an NICU-like experience primes for altered responding to subsequent stressors.

In this study, we show that the development of stress-induced mechanical hypersensitivity after NICU-like experience is successfully disrupted by chemogenetic silencing of the amygdala and perhaps surrounding structures. This implies that for the emergence of mechanical hypersensitivity following an NICU-like experience, activation of the amygdala is necessary during the subsequent adverse event. This finding is supported by other studies highlighting the importance of the amygdala in pain responses (Rouwette et al., 2012). Furthermore, inactivating the amygdala and its central nucleus disrupts pain responses; for example, lesions of the amygdala block the elevated latency of tail flick pain responses (Helmstetter and Bellgowan, 1993), and lesions of the CeA abolish conditioned place aversion in chemical somatic and visceral pain (acetic acid and formalin injections, respectively; Tanimoto et al., 2003) and eliminate shock-induced hyperalgesia and shock-induced sensitization of vocal pain responses to heat (Crown et al., 2000). In addition, activation of CRF1 receptors in the amygdala can trigger pain responses in animals with no actual injury or tissue damage (Ji et al., 2013). We have also previously demonstrated that antagonism of CRF receptors (CRF1 and CRF2) in the CeA during adolescent fear conditioning after an NICU-like experience alters stress-induced hypersensitivity observed in the subjects that experienced early-life injury, with CRF1 antagonism reducing fear-induced hypersensitivity and CRF2 antagonism producing a general antinociceptive effect (Davis et al., 2021). These results demonstrate the implication of amygdala CRF signaling on pain processing and priming enhanced responses to future painful stimuli. Importantly, despite some evidence that the left and right CeA may play opposing roles in pain (Ji and Neugebauer, 2009; Sadler et al., 2017; Allen et al., 2023; Kolber et al., 2010), bilateral inactivation completely blocked our conditioning-induced hypersensitivity in our NICU subjects.

Indeed, within the CeA, the neuromodulator CRF has been implicated in studies of both anxiety and pain for its ability to modulate pain and fear responses (Pomrenze et al., 2019; Mazzitelli et al., 2022; Andreoli et al., 2017; Asan et al., 2005; Chudoba and Dabrowska, 2023; Ji and Neugebauer, 2008). We have previously shown that our NICU-like model drives the expression of CRF and c-fos at the time of the neonatal manipulations and is then followed by a later decrease in CRF-expressing neurons during adolescence (Davis et al., 2021). Given these findings, we anticipated that CeA-CRF neurons may be key in the effects of NICU-like experience on stress-induced hypersensitivity. In Cre-negative subjects (those without CRF cell silencing), we observed a statistically significant difference in withdrawal threshold, with the NICU-like group exhibiting mechanical hypersensitivity compared to non-injected control subjects. As hypothesized, this difference was not observed in Cre-positive subjects (those with CRF silencing), suggesting that chemogenetic silencing of CRF cells may have disrupted the development of hypersensitivity. However, although the inhibition of CeA-CRF neurons at the time of the adolescent fear conditioning disrupted the development of the hypersensitivity typically observed, it also failed to completely reverse it, in that there was not a significant difference between Cre-negative and Cre-positive NICU-exposed subjects. Our observation that stress-induced hypersensitivity following NICU-like experience is impacted by the involvement of CRF neurons in the CeA is consistent with literature pointing toward the role of CeA-CRF cells in chronic pain (Andreoli et al., 2017) and the transition from acute to chronic pain (Kiritoshi et al., 2024). Our results suggest that NICU-like experience may result in a pro-pain state in adolescence that is similar to that observed in the acute stage of neuropathic pain in adults. The fact that CRF cell silencing failed to produce a complete reversal of the hypersensitivity suggests that other cell populations are also likely involved—such as somatostatin and PKCδ-expressing populations (Wilson et al., 2019; Fu et al., 2008). In addition, given the potentially lateralized functions of the CeA in pain (Chen et al., 2022; Wilson et al., 2019; Carrasquillo and Gereau, 2008; Ji and Neugebauer, 2009; Carrasquillo and Gereau, 2007; Sadler et al., 2017; Allen et al., 2023; Chudoba and Dabrowska, 2023), it is possible that silencing CRF cells in both the left and right hemispheres counteracted each other. While we did not see such an effect when the entire amygdala was silenced, it remains possible that only certain populations of CeA cells exhibit lateralized roles in pain, with CRF cells among them. Future work with unilateral silencing could answer this question.

The current data are also consistent with the literature on CeA-CRF cells in anxiety responding and persistent fear after early-life pain, as we observed that inhibition of CRF-expressing cells resulted in decreased auditory freezing (i.e., disrupted conditioned fear) for subjects that endured an NICU-like experience (Victoria and Murphy, 2016a). However, we also observed an increased tone difference score (i.e., heightened conditioned fear response) for subjects not exposed to an NICU-like experience, which was unanticipated. Taken together, these findings highlight a complex role of CeA-CRF neurons that may vary depending on the history of the subject (e.g., previous trauma) in mediating fear responses and the need for further investigation into their involvement in fear processing, especially following early-life trauma.

Hyperexcitability of neurons in the CeA has been linked to both pro- and anti-nociceptive states (Chen et al., 2022; Wilson et al., 2019). For example, Wilson and colleagues demonstrated the amygdala functions as a “pain rheostat,” with opposing injury-induced functions—where, after injury, CeA neurons that express PKCδ are hyperexcitable and demonstrate pronociceptive activity upon stimulation, while those expressing SOM become hypoexcitable, and stimulating them results in antinociceptive behaviors. Neurons in the CeA that express CRF have been shown to be hyperexcitable across a variety of pain conditions (Neugebauer, 2020), and modulation of CRF neurons influences pain sensitivity and anxiety responses (Mazzitelli et al., 2022; Hein et al., 2021; Ji and Neugebauer, 2020; Navratilova et al., 2019; Yakhnitsa et al., 2022). In our study, we show that the neonatal pain endured in our model of an NICU-like experience results in a lasting hyperexcitability of CRF-expressing neurons in the CeA, in that changes to their activity are observable in *ex vivo*, acute brain slice preparations, in the absence of continued input from the animal. The observation that this change persists into later life suggests the development of a pain-related plasticity which could contribute to a pro-pain response to subsequent stress events/injuries that occur during this period. Moreover, neurons within the amygdala display several distinct action potential firing phenotypes that have been observed in a variety of investigations (Lopez De Armentia and Sah, 2004; Chieng et al., 2006; Dumont et al., 2002; Schiess et al., 1999), and this observation holds true for CeA-CRF neurons (Li et al., 2022). Similar to these studies, in our work, the majority of CeA-CRF neurons recorded in this study displayed late-firing action potential phenotypes, although regular spiking, low-threshold bursting, and spontaneously active neurons were also observed. Interestingly, we did not observe spontaneously active CRF neurons under animals in the NICU-like condition. As is also described in published literature, the pain-induced changes to the excitability of CeA neurons observed in this study appear to be driven specifically by late-firing neurons (Wilson et al., 2019; Mazzitelli et al., 2022; Kiritoshi et al., 2024).

There are several limitations to this study that warrant consideration. Due to concerns about the potential impact of neonatal tissue collection in control rodents, Cre status was not determined in our chemogenetic experiments until after the experiments were completed, resulting in disparities in group size, with some experimental groups having as few as five subjects. In contrast, for our electrophysiology experiments, since CRF expression was necessary to obtain recordings from the correct neurons, all animals, even those in the undisturbed condition, underwent neonatal tissue collection for genotype sampling. This intervention, along with the subsequent manipulations for maintaining animal ID markings, introduced potential procedural pain and additional experimenter handling, resulting in an undisturbed condition that is not truly undisturbed. However, the robust increase in excitability observed in the NICU-like condition leads us to believe that this factor did not significantly impact our overall conclusion.

The second largest limitation of this experiment is its exclusion of female subjects. As a population that is greatly impacted by a variety of pain conditions, understanding pain mechanisms in females is of significant research importance. Although we have observed the same behavioral outcomes for heightened pain sensitivity following acute stress in post-NICU rats for both males and females, we did not observe the same changes to CRF in the CeA (Davis et al., 2021; Zuke et al., 2019) and therefore did not include females in the investigation of CeA-CRF cell involvement in stress-induced hypersensitivity following early-life pain. Future studies should therefore investigate this phenomenon in females, perhaps within the hypothalamus, as we have previously observed differences in crh expression and activation in the hypothalamus of females after early-life pain (Zuke et al., 2019) and suspect a similar mechanism of stress-induced hypersensitivity occurs here in females.

While the constitutive DREADD experiment assures us that our DREADDs worked as intended—we see clear behavioral differences in both fear- and pain-related measures—we did not construct any independent verification of the silencing. Given that our CRF-Cre model has been well-validated in expressing Cre in the vast majority of CRF cells in the CeA and not in other populations of cells (Pomrenze et al., 2015), we are confident that the correct cell population was targeted. However, it is possible that silencing was incomplete, especially in the Cre-dependent chemogenetic experiment, as our developmental timeline necessitates implementation and investigation of this expression less than a month after its injection, and we did not conduct any independent verification of the silencing. Furthermore, in the constitutive DREADD experiment, viral spread was not restricted to the CeA, making it possible that inhibiting the surrounding regions contributed to our effect.

Another, lesser limitation of this study was that the electrophysiological investigation of changes after our NICU model only occurred in CeA-CRF neurons, and it is therefore unable to address whether this hyperexcitability is exclusive to CRF-expressing neurons. Based on other published work on pain and anxiety, we would expect that other populations of neurons (such as those that express PKCδ) in the CeA would also be hyperexcitable in our NICU-like experience model. Although other cells may also be hyperexcitable, that does not diminish the importance of the involvement and alterations to CeA-CRF cells after early-life adversity (a previously uninvestigated subject).

As the rate of NICU admissions continues to climb, so does the need to understand the long-term impacts of early-life pain. Taken together, although there are factors that influence interpretation, these data provide strong support for alterations to and involvement of the amygdala, including (but potentially not limited to) cells that express CRF in the central amygdala, in the development of hypersensitivity to pain after an acute stress experience in animals that experienced adversity early in development. We showed that an NICU-like experience alters responding to traumatic experiences in adolescence, such that there is a vulnerability to altered fear responding and trauma-induced hypersensitivity to pain during adolescence, a process at least partially mediated through CeA-CRF neurons. This study also demonstrated CRF's pro-nociceptive role in the CeA, in that our model increased the excitability of CeA-CRF cells, a change that is observed in many other pro-pain states. While we have demonstrated that neurons in the amygdala, including CeA-CRF neurons, contribute to lasting mechanical hypersensitivity following early-life pain and stress, their failure to completely reverse/block the development of stress-induced hypersensitivity suggests other neuronal populations within the amygdala may also play a role, but the specific populations of neurons responsible remain unclear.

In conclusion, neonatal trauma results in a lasting vulnerability to stress-induced mechanical hypersensitivity, with the amygdala playing a key mediating role. This is evidenced by findings that amygdala activation is necessary for the development of hypersensitivity, with partial involvement of CeA-CRF neurons; though further research is needed to clarify the extent of their role.

These findings contribute to the growing body of literature on the long-term impact of early-life adversity, highlighting how the amygdala and CeA-CRF neuronal involvement may underlie persistent vulnerabilities to later adversity stemming from neonatal stress and trauma.

## Data Availability

The raw data supporting the conclusions of this article will be made available by the authors, without undue reservation.
